# Progress in Harmonizing Tiered HIV Laboratory Systems: Challenges and Opportunities in 8 African Countries

**DOI:** 10.9745/GHSP-D-16-00004

**Published:** 2016-09-28

**Authors:** Jason Williams, Farouk Umaru, Dianna Edgil, Joel Kuritsky

**Affiliations:** a Partnership for Supply Chain Management (PFSCM), Supply Chain Management System (SCMS), Arlington, VA, USA. Now with the U.S. Agency for International Development (USAID), Bureau for Global Health, Office of HIV/AIDS, Supply Chain for Health, Washington, DC, USA; b PFSCM, SCMS, Arlington, VA, USA; c USAID, Bureau for Global Health, Office of HIV/AIDS, Supply Chain for Health, Washington, DC, USA

## Abstract

In 2014, the Joint United Nations Programme on HIV/AIDS released its 90-90-90 targets, which make laboratory diagnostics a cornerstone for measuring efforts toward the epidemic control of HIV. A data-driven laboratory harmonization and standardization approach is one way to create efficiencies and ensure optimal laboratory procurements. Following the 2008 “Maputo Declaration on Strengthening of Laboratory Systems”—a call for government leadership in harmonizing tiered laboratory networks and standardizing testing services—several national ministries of health requested that the United States Government and in-country partners help implement the recommendations by facilitating laboratory harmonization and standardization workshops, with a primary focus on improving HIV laboratory service delivery. Between 2007 and 2015, harmonization and standardization workshops were held in 8 African countries. This article reviews progress in the harmonization of laboratory systems in these 8 countries. We examined agreed-upon instrument lists established at the workshops and compared them against instrument data from laboratory quantification exercises over time. We used this measure as an indicator of adherence to national procurement policies. We found high levels of diversity across laboratories’ diagnostic instruments, equipment, and services. This diversity contributes to different levels of compliance with expected service delivery standards. We believe the following challenges to be the most important to address: (1) lack of adherence to procurement policies, (2) absence or limited influence of a coordinating body to fully implement harmonization proposals, and (3) misalignment of laboratory policies with minimum packages of care and with national HIV care and treatment guidelines. Overall, the effort to implement the recommendations from the Maputo Declaration has had mixed success and is a work in progress. Program managers should continue efforts to advance the principles outlined in the Maputo Declaration. Quantification exercises are an important method of identifying instrument diversity, and provide an opportunity to measure efforts toward standardization.

## Abstract (precis)

Countries have had mixed results in adhering to laboratory instrument procurement lists, with some limiting instrument brand expansion and others experiencing substantial growth in instrument counts and brand diversity. Important challenges to advancing laboratory harmonization strategies include:

1. Lack of adherence to procurement policies
2. Lack of an effective coordinating body
3. Misalignment of laboratory policies, treatment guidelines, and minimum service packages

## INTRODUCTION

In 2014, the Joint United Nations Programme on HIV/AIDS (UNAIDS) released targets of testing 90% of people living with HIV/AIDS, placing 90% of those with HIV/AIDS on antiretroviral therapy, and ensuring that 90% of those on antiretroviral therapy are virally suppressed.^1^ These 90-90-90 targets made laboratory diagnostics a cornerstone for national efforts toward the epidemic control of HIV. A data-driven laboratory harmonization and standardization approach is one way to create efficiencies and ensure optimal laboratory procurements.

In 2008, a consensus meeting on clinical laboratory testing, harmonization, and standardization was held in Maputo, Mozambique. Representatives of governments, multilateral agencies, development partners, professional associations, and academic institutions sought to address overarching laboratory challenges that had limited the scale-up of services for tuberculosis, malaria, and HIV diagnosis and care.^2^ The meeting was organized by the World Health Organization | Regional Office for Africa (WHO-AFRO) and the U.S. President’s Emergency Plan for AIDS Relief (PEPFAR), with the support of the World Bank, The Global Fund to Fight AIDS, Tuberculosis and Malaria, the Bill & Melinda Gates Foundation, the Clinton Health Access Initiative (CHAI), and the Partnership for Supply Chain Management (PFSCM). The outcome of this meeting was the “Maputo Declaration on Strengthening of Laboratory Systems”: a call to governments to take leadership in harmonizing tiered laboratory networks and standardizing testing services.^2^

Those attending the meeting—120 experts and policy makers from 33 countries, including representatives from 28 sub-Saharan African countries—were invited to reach a consensus on technical and operational guidance for strategic planning for responsive laboratory development. Participants recognized a need to address the challenges limiting the uptake of diagnostic services in resource-limited settings. These challenges included lack of or insufficient leadership and advocacy, human resources, national laboratory policies, strategic and financial planning, physical infrastructure, supply chain management, and quality management systems.^2^

**FIGURE 1.**
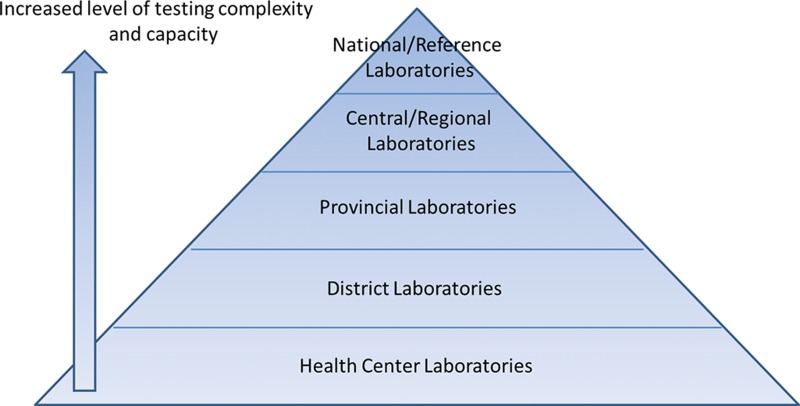
Example of a Hierarchical Tiered Laboratory System Adapted from the 2008 Maputo Declaration.^2^

To address these issues, participants recommended that countries adopt a tiered laboratory system strategy within a harmonized network. A tiered laboratory system features stratified levels of laboratories (national, central/regional, provincial, district, and health center) based upon agreed testing services, with each level offering increased technical testing complexity and capacity (Figure 1). This tiered laboratory scheme is critical to strengthening public health laboratory services and informing effective national laboratory policy.^3^^,^^4^

A tiered laboratory system, within a harmonized network, is critical to strengthening public health laboratory services.

In addition to the call for government leadership in the Maputo Declaration, the meeting resulted in technical and operational recommendations to guide the harmonization and standardization of clinical laboratory testing in developing countries. Key recommendations from group breakout sessions included the following:

- Prioritize laboratory system coordination by developing national laboratory policies, establishing departments of laboratory systems within ministries of health, and calling upon donors and partners to support national governments in this effort.
- Define and establish the minimum test offerings required at each level of an integrated, tiered laboratory network, as well as the associated diagnostic instruments, equipment, and human resources required to provide such services.
- Prioritize supply chain systems and maintenance and service contracts for laboratory-based equipment at all levels of the laboratory network.

The 2008 Maputo Declaration recommended prioritizing coordination of national laboratory systems.

Following the 2014 Ebola outbreak in Western Africa, a follow-on harmonization meeting was held in Freetown, Sierra Leone, in October 2015. This meeting extended the call for international and local laboratory partners to increase capacity and further emphasized the need to develop tiered laboratory networks. The Freetown meeting brought together the African Society for Laboratory Medicine, WHO-AFRO, and ministry of health officials from more than 20 countries in Africa. The meeting resulted in the “Freetown Declaration on Developing Resilient Laboratory Networks for the Global Health Security Agenda in Africa,” which announced the need to effectively integrate tiered laboratory networks into disease surveillance and public health institutes.^5^ The declaration also emphasized the need to regularly measure progress with a standardized scorecard.^6^ The recent Ebola outbreak clearly demonstrates the critical need to reduce vulnerabilities in health care facilities and the laboratory system interface.

## ESTABLISHING A STRATEGY FOR LABORATORY HARMONIZATION AND STANDARDIZATION

From 2008 to 2015, expenditures for laboratory instruments and commodities have increased substantially among all countries, with the aim of addressing access to critical HIV-related laboratory services. PEPFAR, The Global Fund, CHAI, and others have led efforts to expand coverage of diagnostic instrumentation as part of the global response to the HIV epidemic. Between 2007 and 2016, the United States Agency for International Development’s (USAID’s) primary PEPFAR procurement mechanism was PFSCM’s Supply Chain Managment System. USAID’s financial contribution through this mechanism toward instrument procurements and laboratory commodity requirements increased from US$33,759,096 (2008) to $82,152,562 (2015) following the Maputo Declaration, for a total contribution of $511,475,320 across 43 countries.^7^ Countries have introduced hundreds of diagnostic instruments to reach patients within their laboratory networks, as well as at the health center level with the introduction of point-of-care (POC) instrumentation.

Now 8 years after Maputo, we review in this article how this financial and technical support has enhanced efforts to implement harmonization strategies as part of scale-up efforts. The terms “harmonization” and “standardization” are often used interchangeably among laboratory practitioners and policy makers. Here we define “laboratory harmonization” as a process of coordinating host country governments and stakeholders in the procurement and placement of laboratory products within a defined tiered laboratory network. This process is informed through consultation with key stakeholders, such as physicians, program leads, laboratory professionals, and procurement officers to develop technical policies. We define “standardization” as the process of implementing and adhering to the established technical policies.

Standardization is the process of implementing and adhering to established technical policies.

Laboratory harmonization is the process of coordinating governments and stakeholders in a defined tiered laboratory network.

Harmonization and standardization efforts offer considerable benefits. In South Africa, an integrated and standardized tiered service delivery model for CD4 (cluster of differentiation 4) testing could improve turnaround times by ensuring appropriate placement and integration of POC technologies within the conventional tiered laboratory structure. These efforts demonstrated a reduction of R125 million (US$8.8 million) in HIV/AIDS program costs annually.^8^ Harmonization and standardization also offer the following broader benefits to laboratory service delivery^9^:

- Establishing minimum diagnostic test offerings and standardized testing methods within the tiered health network.
- Reducing variation in laboratory products across different facilities, thereby improving commodity logistic systems, standardized quality control practices, and quality assurances.
- Simplifying the identification and quantification of laboratory-based products.
- Training laboratory staff more efficiently.
- Improving coordination in laboratory instrument procurement, maintenance, and placement practices.

Laboratory harmonization and standardization offer many benefits, including reduced costs and improved turnaround times.

Over the past 9 years, PFSCM’s Supply Chain Management System, a project funded by PEPFAR and administered by USAID, facilitated laboratory harmonization and standardization workshops in 7 African countries at the request of the respective ministries of health. The USAID | DELIVER PROJECT and U.S. Centers for Disease Control and Prevention (CDC) with PEPFAR provided direct assistance for an eighth workshop. These workshops were held in a mix of PEPFAR-supported sub-Saharan African countries (Eastern [3], Western [2], and Southern [3] African countries). In general, requests for workshops were initiated to address HIV supply chain challenges (e.g., quantification, procurement, commodity diversity, and logistics) as well as suboptimal instrument placement and high levels of instrument diversity related to increasing numbers of donated instruments for rapid scale-up of HIV programs.^10^

Facilitated laboratory harmonization and standardization workshops were held in 8 sub-Saharan African countries.

Each harmonization and standardization workshop had these overall objectives:

1. Arrive at consensus on the methodology for harmonization and standardization.
2. Establish national minimum test offerings and methodologies to be employed at each laboratory tier for each test.
3. Derive an evidence-based list of harmonized diagnostic instruments to support the required laboratory services.
4. Establish the minimum ancillary equipment requirements at each tier.
5. Define the staffing complement required at each tier to support the recommended laboratory services.
6. Determine a strategic implementation plan, with defined roles and responsibilities.

A technical consultation document released following the 2008 Maputo meeting was used as a reference standard to initiate workshops. This document details a notional list of tiered test offerings, diagnostic instruments, and ancillary equipment, as well as human resource templates, that countries can use as a starting point to guide their laboratory harmonization efforts.^11^ These lists were generalized to better serve multicountry planning efforts, with detailed recommendations relevant to workshop participants.

**FIGURE 2.**
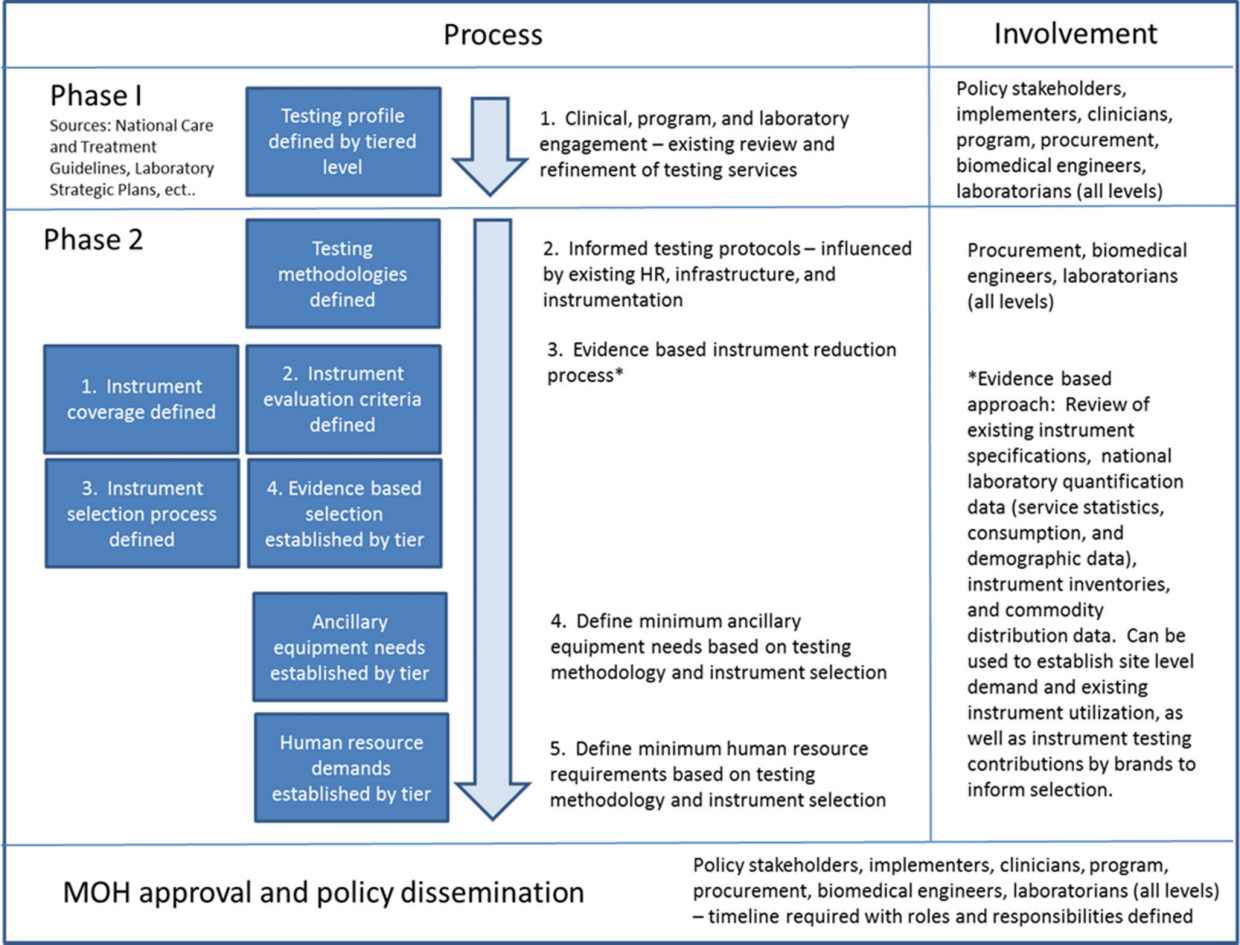
An Evidence‐Based 2‐Phase Approach to Developing a Harmonization and Standardization Proposal

We view facilitation of harmonization and standardization workshops as a 2-step process. Phase 1, at the start, is for policy stakeholders, implementers, clinicians, and key program, procurement, and laboratory staff to define what testing services are required within the health system and at what tier of the laboratory system they should be offered (Figure 2).

In Phase 2, laboratory experts establish the appropriate diagnostic methods to be used for testing services at each tier. The experts then develop a proposed harmonized list of diagnostic instruments by tier, the necessary ancillary equipment, and the staffing required for the defined testing menu. These lists are then translated into a national harmonization and standardization policy for implementation. The instrument harmonization approach is informed by existing coverage of diagnostic instruments and the degree of instrument diversity. Standardized instrument lists should not be limited to one particular brand, but should include several brands to disperse risk across diagnostic specialties and eliminate the potential for monopolization.

## METHODOLOGY FOR REVIEWING PROGRESS IN HARMONIZATION AND STANDARDIZATION

Past evaluations associated with implementation of the Maputo Declaration have been limited. A review of previous work on laboratory harmonization implementation focused on selection of the most appropriate tests and equipment types within the clinical cascade, as well as on how tiered networks are defined.^12^ Other evaluations have reviewed published reports, interviewed donors, and assessed coordination efforts, with implementation of national laboratory plans found to be inconsistent and frequently problematic.^13^ Past evaluations have not targeted instrument brand diversity as a measure of adherence to standardized procurement policies.

Recognizing these limitations, we sought to measure implementation progress over time in the 8 countries in which harmonization and standardization workshops were held. We did this by analyzing available annual HIV laboratory quantification data. These data include instrument types and brands as a component of commodity forecasting over time. We organized standardized data import templates from ForLab (http://www.forlabtool.com), which is a multimethod laboratory forecasting tool, developed in partnership with USAID and CHAI. Laboratory instrument data were extracted by country for multiple forecasting periods. These data would help measure adherence to instrument procurement practices against established harmonization and standardization instrument policies in the countries where we held workshops.

We sought to measure progress in harmonization and standardization over time in 8 countries by analyzing annual HIV laboratory quantification data.

All quantification data used in this comparison were collected through site visits, implementing partner data collection efforts, national equipment inventory lists, and commodity distribution data from national logistics systems. The final instrumentation network was validated in coordination with each country’s national laboratory leadership, as well as by PEPFAR implementing partners and U.S. Government missions (USAID and CDC), before we initiated the national forecasting exercises within ForLab. We performed additional validation at the conclusion of each national laboratory quantification exercise for commodity budgeting and procurement purposes. Additionally, we assessed initial harmonization and standardization proposals for potential instrument reductions by comparing existing diagnostic instrument variety against proposals that were developed at the time of each harmonization and standardization workshop (where data were available). When our harmonization workshops were held, attendees identified recurring challenges to implementing harmonization proposals. These challenges were reviewed to identify obstacles to address as part of implementing harmonization and standardization efforts.

In workshops, attendees identified challenges to address when implementing harmonization and standardization efforts.

The intent of this analysis is (1) to illustrate the efforts made to conduct harmonization and standardization workshops to influence laboratory development; (2) to determine how well these countries have done; and (3) to describe what potential underlying challenges must be overcome to advance laboratory harmonization and standardization efforts.

## FINDINGS

### Instrument Counts and Increased Capacity

**FIGURE 3.**
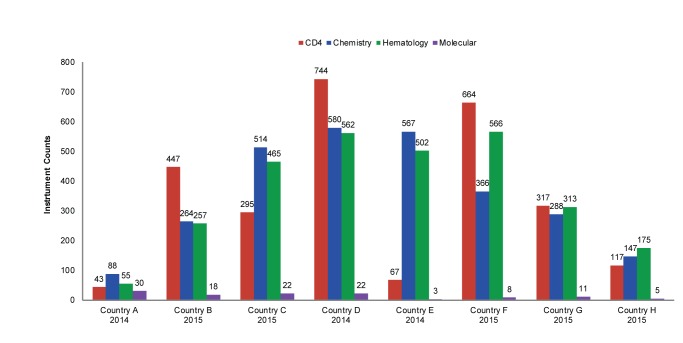
Instrument Counts in 8 African Countries, by Diagnostic Area

**FIGURE 4.**
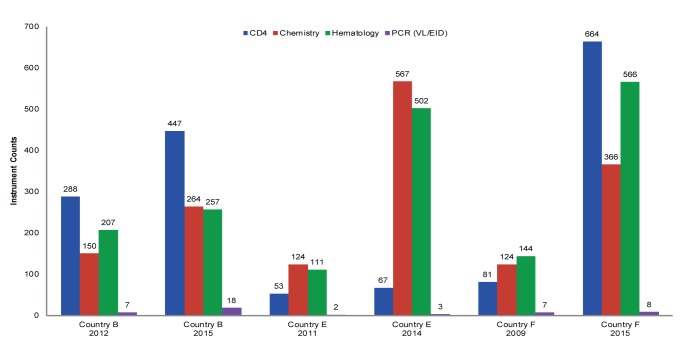
Growth in Instrument Counts in 3 African Countries, by Diagnostic Area, 2009–2015 Abbreviations: EID, early infant diagnosis; PCR, polymerase chain reaction; VL, viral load.

Figure 3 provides a summary of recent instrument counts by country. These numbers were extracted from national HIV laboratory forecasting exercises conducted in 2014 and 2015. Figure 4 represents the percentage growth by diagnostic area over time in the 3 countries where consecutive data points were available (Country B, Country E, and Country F). These 3 countries have had marked increases in instrument counts: Country B has increased CD4 instrumentation by 155% (from 288 to 447) since 2012 by introducing the Becton Dickinson FACSPresto in 2015 to replace aged FACSCounts machines and to expand CD4 testing to lower-level health facilities. From 2011 to 2014, Country E’s chemistry and hematology coverage increased more than 450% (from 124 to 567, chemistry, and from 111 to 502, hematology) due to program scale-up. And Country F reached an 820% growth in CD4 instrumentation (from 81 to 664) since 2009, primarily due to national deployment of Alere Pima CD4 testing machines as a POC solution to address CD4 sample referral challenges.

### Instrument Brand Diversity

**FIGURE 5.**
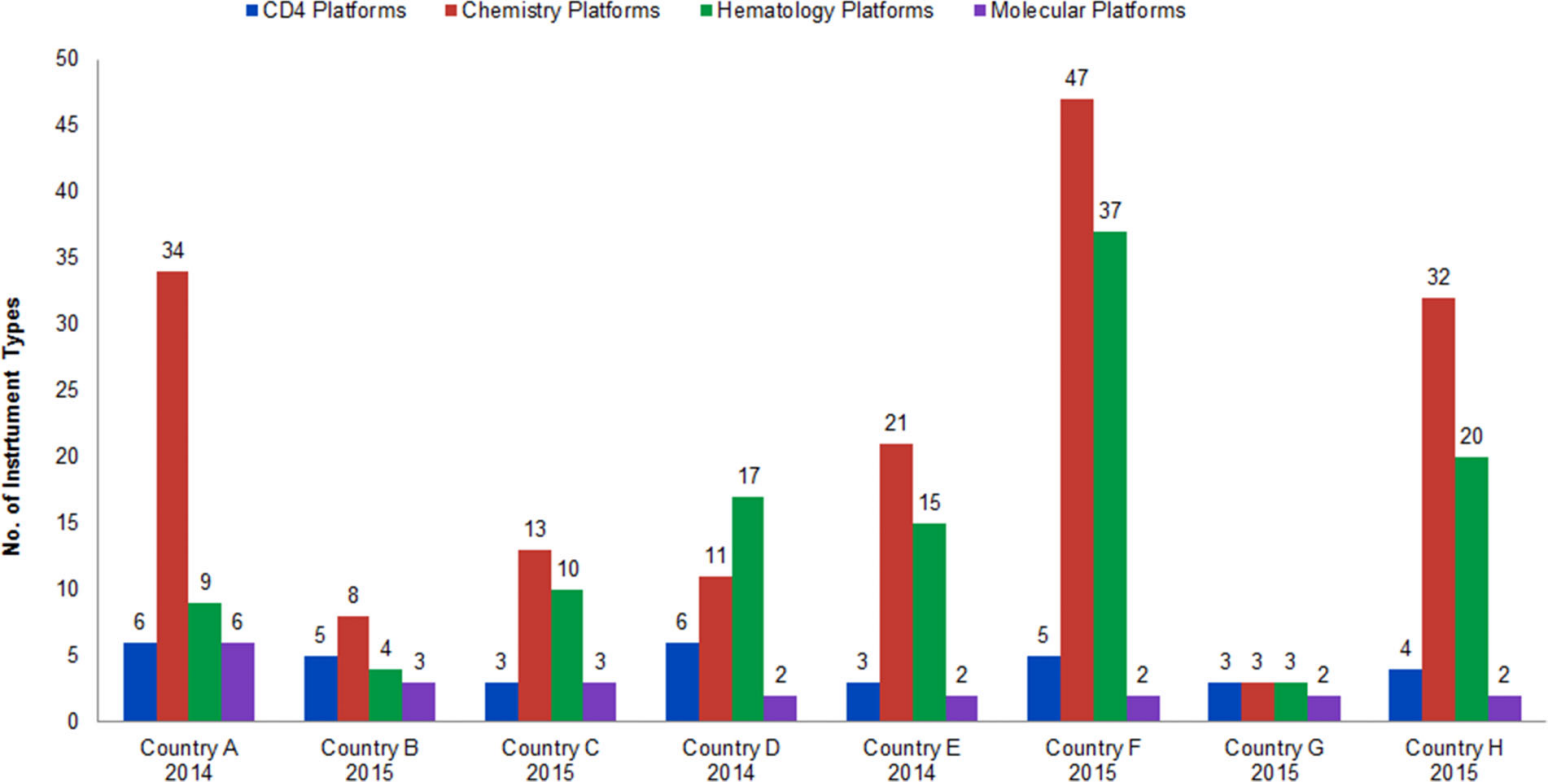
Diversity of Instrument Types in 8 African Countries, by Diagnostic Area

The highest levels of instrument diversity, in manufacturer brand or unique diagnostic instrumentation types, were found in chemistry and hematology (Figure 5). Chemistry and hematology instruments are used in general care and clinical patient management services, but also play a key role in monitoring those on lifesaving HIV treatment. It should be noted that CD4 monitoring and molecular diagnostic instrumentation are predominantly procured through donor mechanisms. These procurements are influenced by the WHO prequalification process for introducing new diagnostic technology and therefore appear more harmonized due to fewer choices of approved brands.^14^^,^^15^

As with CD4 testing instrumentation, glucometers and hemoglobinometers (POC devices) contribute to high instrument counts, but unlike CD4 instrumentation, these devices also contribute to high levels of instrument diversity, with ministries, donors, implementing partners, and other stakeholders procuring many brands. High brand diversity further adds to higher levels of unique commodity types. For example, a CD4 test run on a FACSCount requires a minimum of 6 different items: CD4 reagents, a control kit, clean solution, rinse solution, FACSFlow sheath fluid, and thermal paper. Thus, if a country has 5 different types of CD4 instruments, more than 30 different commodities may be required for CD4 testing alone.

A CD4 test run on a FACSCount requires a minimum of 6 different items; thus, if a country has 5 types of CD4 instruments, more than 30 different commodities may be required for CD4 testing alone.

Many countries are using multiple open systems (e.g., systems in which reagents and consumables are nonproprietary) for chemistry instrumentation, which helps reduce commodity variation by allowing for sharing of reagents and general consumables, but it introduces challenges in training and variation with instrument maintenance. Hematology is a closed system market (e.g., the systems require proprietary reagents), and these systems require many commodity types to keep instruments operational.

### Proposed Reductions in Instrument Diversity

**FIGURE 6.**
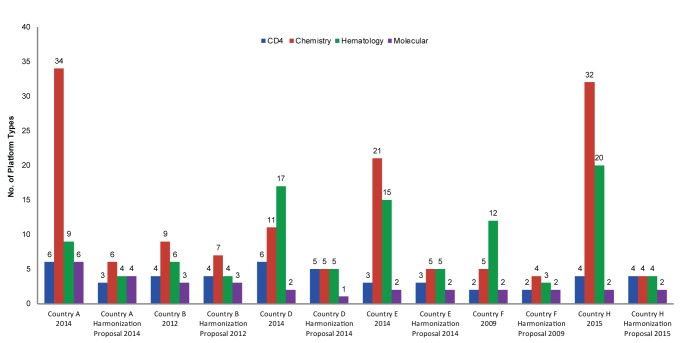
Comparison of Current and Proposed Instrument Diversity in 6 African Countries With Harmonization Proposals

**FIGURE 7.**
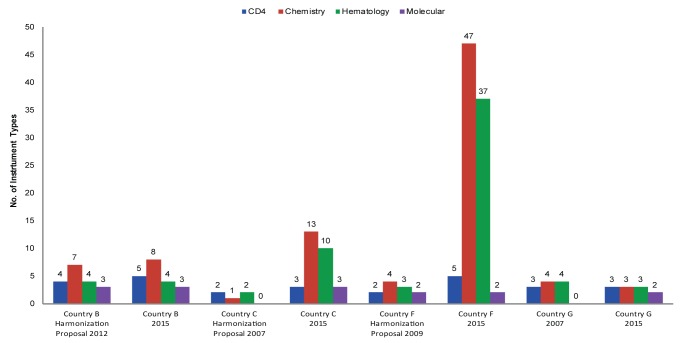
Shifts in Instrument Diversity in 4 African Countries Following Their Harmonization Proposals Note: Country C has 75 additional instruments for chemistry and 55 additional instruments for hematology that are not included in the bar chart.

Following HIV harmonization and standardization workshops, participants proposed substantial reductions in instrument diversity, ranging from a 17% reduction in Country D’s existing CD4 instruments to a high of 88% for Country H’s chemistry testing instruments (Table). As mentioned earlier and demonstrated in Figure 6 and Figure 7, chemistry and hematology account for the most diagnostic instrument diversity, hence have the largest potential for instrument reduction. Potential reduction for both CD4 instrumentation and polymerase chain reaction–based molecular instrumentation is between 1 and 2 from an absolute count perspective.

**TABLE:** Proposed Instrument Reductions by Instrument Type

Country	Proposed Instrument Reduction			
	CD4	Chemistry	Hematology	Molecular
Country A	-50%	-82%	-56%	-33%
Country B	0%	-22%	-33%	0%
Country C	N/A	N/A	N/A	N/A
Country D	-17%	-55%	-71%	-50%
Country E	0%	-76%	-67%	0%
Country F	0%	-20%	-75%	0%
Country G	N/A	N/A	N/A	N/A
Country H	0%	-88%	-80%	0%

Note: Percentage reduction cannot be calculated for Country C and Country G due to lack of data on instrument diversity in these countries before harmonization efforts began.

### Efforts to Implement Harmonization and Standardization

We compared the earlier adopters of the Maputo Declaration—Country B (2012), Country C (2007), Country F (2009), and Country G (2007)—and found notable differences between countries in implementation and adherence to harmonization strategies (Figure 7).

Different countries had different levels of implementation and adherence to harmonization strategies.

Country B and Country G have been successful at limiting instrument brand expansion. Country B’s success is partly due to PEPFAR, which has historically provided funding for and coordinated closely with partners and the ministry of health around procuring laboratory instrumentation with the goal of complying with standardized instrument lists. Country G has succeeded by limiting commodity availability and supporting procurement and distribution of commodities only for approved instrumentation. Both of these countries have improved national laboratory forecasting efforts, which are now led by national quantification committees and have directly influenced commodity availability and improved laboratory logistic system proficiency due to reduced commodity counts. For example, as part of its harmonization efforts, Country G designed an initial laboratory logistics system in 2007 that reduced commodity types from more than 400 down to 185 HIV-specific products. Once the national logistics system was established and institutionalized, the commodity profile was later expanded to include a full array of diagnostic products (more than 380), further expanding the breadth of services offered and improving the overall planning and procurement practices associated with laboratory service delivery overall, not just HIV-related services.^16^^,^^17^

National laboratory quantification committees can directly influence commodity availability and improve laboratory logistic systems.

Conversely, Country C and Country F appear to have had less success in implementing an approach that would reduce instrument diversity, and instead have experienced substantial growth in brand diversity. Country F has seen marked increases in chemistry and hematology instrument diversity, much of which was driven by POC technology, with molecular instrumentation being the only diagnostic area that has remained constant. As stated earlier, in many countries the coordination efforts associated with implementing national laboratory plans and adherence to harmonization and standardization policies have been inconsistent and frequently problematic.^13^^,^^18^ We believe that this may play an important role in the ability of Country C and Country F to achieve leveled or decreasing brand diversity.

Another 4 of the evaluated countries—Country A, Country D, Country E, and Country H—have developed harmonization and standardization proposals only within the last 3 years, so it is difficult at this point to determine how well these countries will succeed at advancing their procurement practices to better align to their proposed strategies.

### Common Challenges and Critical Success Factors to Advancing Harmonization

Throughout the harmonization and standardization efforts facilitated by PFSCM’s Supply Chain Management System, in-country program leads, stakeholders, and workshop participants expressed recurring challenges in advancing harmonization strategies. Overall, 10 recurring challenges were identified across countries: the structure of and lack of adherence to existing procurement policies, misalignment of service delivery policies and guidelines, lack of defined laboratory tiers, lack of an effective coordinating body responsible for laboratory harmonization, and issues with equipment maintenance, data availability, managing frequent shifts in technology, human resources, competing priorities, and political agendas. To advance the harmonization and standardization agenda at a national level, we believe that 3 of the challenges identified by participants are the most important to address: (1) lack of adherence to procurement policies (i.e., instrument diversity), (2) lack of an effective coordinating body, and (3) misalignment of laboratory policies, treatment guidelines, and minimum services.

Across countries, 10 recurring challenges to laboratory harmonization have been identified.

#### Lack of Adherence to Procurement Policies

There are high levels of instrument diversity in laboratory diagnostic instruments, equipment, and services across health facilities. The lack of coordination in the procurement and deployment of laboratory equipment contributes to different levels of compliance with service delivery standards across facilities that provide similar levels of care.

Compounding this challenge, procurement agents and national governments may see reducing instrument diversity as reducing competitiveness. They may assume that limiting the number of types of diagnostic instruments will lead to sole and single sourcing of instruments and reagents, creating monopolies and restricting competition, which contradicts most country-level procurement regulations.

If harmonization and standardization policies were static, this argument could hold true, but policies must be dynamic and based on instrument and vendor performance. In addition, over time, systems and clinical demands shift, technology advances, and existing instruments age and become obsolete. Countries thus must update standardized instrument lists to align service delivery expectations and ensure that laboratories can provide the necessary services with instruments that perform well, with reliable vendor support. Monitoring instrument and vendor performance should be continuous. These performance measures have traditionally been linked to commodity logistics systems, which are based on procurement lead times and instrument repair response times that inform replenishment of reagents. Historically, these systems have had challenges, but with meaningful technical support and investment from PEPFAR, commodity logistics systems are improving. Harmonization and standardization policy reviews should occur at a minimum of every 2 years, and should use annual laboratory quantification and logistics data to measure progress and general instrument and vendor performance, but also to assess the potential for introducing new technology.

Harmonization and standardization policies must be dynamic and based on instrument and vendor performance.

#### Lack of an Effective Coordinating Body

Many countries do have a national laboratory directorate or coordinating body responsible for guiding laboratory development, but they are unable to prioritize laboratory harmonization and move the harmonization agenda from proposal to actual policy. This may be due to national priorities or political agendas, with laboratory technical working groups operating without a formal mandate or authority. Formalizing laboratory technical working groups with specific terms of reference, authority, and accountability would support advocacy efforts and help finalize harmonization and standardization proposals, as well as guide implementation.

Additionally, technical working groups should be charged with monitoring instrument procurement and placement, as well as laboratory technology development. This will ensure that ministries of health define processes for evaluating new technologies before they are deployed. The working groups should also guide ministries in developing policy for laboratory network development and other national laboratory interests. Once policy is finalized by the technical working groups, it is critical to ensure stakeholder adherence to standardization of procurement practice, as well as instrument placement.

Technical working groups should be charged with monitoring instrument procurement and placement, as well as laboratory technology development.

#### Misalignment of Laboratory Policies, Treatment Guidelines, and Minimum Services

In many countries, laboratory policies, minimum packages of care, and national HIV/AIDS care and treatment guidelines are not aligned at the time of harmonization and standardization workshops. Laboratory and treatment policies and guidelines may have been updated with differing frequencies and without coordination between laboratory staff and clinicians. Many laboratories may have been providing tests that were outdated or not aligned with the minimum care needs by tier, or tests that were not clearly defined within existing laboratory policy and/or strategy documents.

Laboratories may provide tests that are outdated or not aligned with the appropriate tier’s minimum care needs.

For example, the harmonization and standardization effort in Country A began with a review of the country’s laboratory strategic plan, the essential health service package, the integrated health service plan, and the HIV/AIDS care and treatment guidelines. In Country E, a policy review was initiated with the country’s norms and standards for medical laboratories, the laboratory strategic plan, and the HIV/AIDS prevention and treatment guidelines. When long-standing strategic plans cover many years and HIV treatment guidelines are more dynamic, these documents can quickly diverge in regard to priorities, implementation planning, and overall expectations associated with laboratory service delivery.

Additionally, budget growth and scale-up efforts for laboratory, program, and clinical needs increasingly diverge, further widening the gap between clinical needs and laboratory service capacity.

In most cases, the harmonization and standardization workshops were the first time clinicians and program staff had met in a large forum to discuss laboratory service delivery challenges, to define minimum test offerings by tier, and to advance a coordinated and aligned way forward.

To address these challenges, it is important to ensure that when treatment guidelines or minimum packages of care are updated, laboratory personnel have the opportunity to inform decision makers of existing laboratory capacity and scalability. Laboratory, clinician, and program staff should be engaged frequently to ensure that the laboratory network evolves to meet clinical and program needs. Laboratory investigations should be fully integrated into clinical and preventive protocols and programs, with the rational use of essential tests relevant to the level and type of facility.

As consumers of laboratory tests, clinicians should help laboratory programs determine test offerings for each laboratory tier based on the established national health care package, clinical importance, cost, suitability to the environment, and level of expertise of the service provider and end users.

Tests offered at each laboratory tier should be based on the established national health care package, clinical importance, cost, suitability, and level of provider expertise.

### Limitations

We recognize several limitations to the analysis described here. Our current analysis targets HIV-related diagnostics only. This choice was due to the high quality of HIV laboratory data that was available. It should be noted that all harmonization and standardization workshops included all diagnostic services and were not limited to just HIV diagnostics.

Our analysis targets HIV-related diagnostics only, due to the high quality of available data.

The primary measure of harmonization and standardization in this article was limited to instrument brand diversity, as a way to measure compliance to procurement from a national standardized instrument list. Other evaluations have sought to focus on appropriateness of tests and equipment types within the clinical cascade, as well as on how tiered networks are defined and implemented.^12^^,^^13^ Ideally, a combination of measures associated with testing availability within the laboratory network, instrument brands, and potential placement of instruments within the tiered laboratory network would provide a more complete picture of harmonization and standardization success. This could even provide opportunities to identify optimization strategies that build upon laboratory standardization efforts. Additional research could provide greater understanding of which components of harmonization efforts have achieved success and which are works in progress or more challenging to implement more broadly.

Implementing a harmonization and standardization policy and demonstrating alignment to a standardized instrument list can take many years. Half of the countries included in this analysis had completed harmonization workshops before 2013, the other half completed their workshops more recently. A follow-up evaluation in a few years could identify additional challenges not considered here, or could demonstrate further compliance and success.

## CONCLUSION

The Maputo Declaration and, more recently, the Freetown Declaration, called on national governments to prioritize laboratory system development and emphasized the need to foster national ownership; more importantly, participants at these two meetings urged donors and partners to commit to working in close coordination to support efforts to strengthen sustainable public health laboratory systems.

Although the list of tiered test offerings and diagnostic instruments included in the Maputo Declaration^11^ is now dated, the founding principles and primary objectives are still relevant today, as illustrated by the 2015 Freetown Declaration. New technologies are emerging to address diagnostic and patient-monitoring challenges, along with additional levels of complexity in laboratory networks due to the introduction of POC technology and associated decentralization of laboratory services. A strategic harmonization and standardization framework is critical to ensure a coordinated and sustainable laboratory development agenda.

Overall, harmonization and standardization efforts have been implemented with mixed success, with some countries only recently implementing measures to introduce harmonization policies. Effectively implementing a harmonization and standardization policy and demonstrating alignment to a standardized instrument list can take many years. For example, the rate of replacing existing equipment with approved instruments depends on instrument life span. Using harmonization and standardization principles as programs scale up, with new instruments purchased for replacements and network expansion at new sites, will limit growth in instrument and brand diversity. Leveraging scorecards, as recommended by the Freetown Declaration, may be another way to advance these efforts.

Since the inception of the global response to the HIV epidemic, procurements of HIV-related diagnostic instruments have increased markedly. A data-driven laboratory harmonization and standardization approach is one way to ensure optimal use of laboratory-based instrument diagnostics and to create efficiencies in product procurement, placement, training, and use. This will be increasingly important to efforts to achieve UNAIDS’ 90-90-90 targets.^1^

However, less has been done to regulate procurement and reduce instrument diversity within chemistry and hematology, where critical safety monitoring tests for antiretroviral therapy are performed, or to provide highly needed general health and screening diagnostics. As a result, chemistry and hematology now represent the highest levels of instrument diversity within laboratory networks.

A high level of instrument diversity has a great impact on laboratory commodity forecasting, supply chain systems, equipment maintenance, and quality laboratory service delivery. Although many years have passed since the Maputo meeting in 2008, the 2015 Freetown Declaration illustrates the continued need to improve adherence to harmonization and standardization practices. Improved coordination is required, as well as the development, implementation, and—more importantly—monitoring and updating of laboratory harmonization and standardization policies over time. Developing a tiered laboratory network, procuring from standardized instrument lists, as well as ensuring the alignment of laboratory policies, minimum packages of care, and national care and treatment guidelines, are all critical to achieving the benefits of harmonization and standardization.

As donor contributions and priorities shift in response to viral load scale-up as part of the 90-90-90 effort among HIV practitioners, there is potential for less support and focus on chemistry and hematology instrumentation, as well as on other diagnostic specialties. Support for HIV programs has historically included procurement of chemistry- and hematology-related commodities and instruments, with the assumption that local governments will assume responsibility for these lower-cost tests moving forward. It is therefore critical to improve efforts to ensure that national ministries of health can expand and sustain not only their existing HIV-related laboratory services but also the general public health laboratory services, to serve broader health and surveillance needs. It is important for program managers, supply chain activity managers, and others to work with their colleagues to understand the capacity, use, and placement of instrument-based diagnostics. There is still a need to reduce excess numbers of products, improve use, decrease costs, and increase efficiency.

Important gains have already been achieved within national laboratory networks, but there is still a need to ensure that countries are able to provide continued quality laboratory services in an efficient and sustainable manner. The Maputo and Freetown Declarations provide a detailed strategic approach that is critical to ensure a coordinated and sustainable laboratory development agenda to address the broader health security agenda in Africa.

## Notes

**Peer Reviewed**
